# The identities of insulin signaling pathway are affected by overexpression of Tau and its phosphorylation form

**DOI:** 10.3389/fnagi.2022.1057281

**Published:** 2022-12-16

**Authors:** Ningtian Ma, Yuyang Liang, Lingyun Yue, Pu Liu, Yuxia Xu, Cuiqing Zhu

**Affiliations:** State Key Laboratory of Medical Neurobiology and MOE Frontiers Center for Brain Science, Institutes of Brain Science, Fudan University, Shanghai, China

**Keywords:** Tau, pseudo-phosphorylation, insulin signaling pathway, lonafarnib, Alzheimer’s disease

## Abstract

**Introduction:**

Hyperphosphorylated Tau formed neurofibrillary tangles was one of the major neuropathological hallmarks of Alzheimer’s disease (AD). Dysfunctional insulin signaling in brain is involved in AD. However, the effect of Tau pathology on brain insulin resistance remains unclear. This study explored the effects of overexpressing wild-type Tau (WTau) or Tau with pseudo-phosphorylation at AT8 residues (PTau) on the insulin signaling pathway (ISP).

**Methods:**

293T cells or SY5Y cells overexpressing WTau or PTau were treated with or without insulin. The elements in ISP or the regulators of IPS were analyzed by immunoblotting, immunofluorescent staining and co-immunoprecipitation. Akt inhibitor MK2206 was used for evaluating the insulin signaling to downstream of mTOR in Tau overexpressing cells. The effects of anti-aging drug lonafarnib on ISP in WTau or PTau cells were also analyzed with immunoblotting. Considering lonafarnib is an inhibitor of FTase, the states of Rhes, one of FTase substrate in WTau or PTau cells were analyzed by drug affinity responsive target stability (DARTS) assay and the cellular thermal shift assay (CETSA).

**Results:**

WTau or PTau overexpression in cells upregulated basal activity of elements in ISP in general. However, overexpression of WTau or PTau suppressed the ISP signaling transmission responses induced by insulin simulation, appearing relative higher response of IRS-1 phosphorylation at tyrosine 612 (IRS-1 p612) in upstream IPS, but a lower phosphorylation response of downstream IPS including mTOR, and its targets 4EPB1 and S6. This dysregulation of insulin evoked signaling transmission was more obvious in PTau cells. Suppressing Akt with MK2206 could compromise the levels of p-S6 and p-mTOR in WTau or PTau cells. Moreover, the changes of phosphatases detected in WTau and PTau cells may be related to ISP dysfunction. In addition, the effects of lonafarnib on the ISP in SY5Y cells with WTau and PTau overexpression were tested, which showed that lonafarnib treatment resulted in reducing the active levels of ISP elements in PTau cells but not in WTau cells. The differential effects are probably due to Tau phosphorylation modulating lonafarnib-induced alterations in Rhes, as revealed by DARTS assay.

**Conclusion and discussion:**

Overexpression of Tau or Tau with pseudo-phosphorylation at AT8 residues could cause an upregulation of the basal/tonic ISP, but a suppression of insulin induced the phasic activation of ISP. This dysfunction of ISP was more obvious in cells overexpressing pseudo-phosphorylated Tau. These results implied that the dysfunction of ISP caused by Tau overexpression might impair the physiological fluctuation of neuronal functions in AD. The different effects of lonafarnib on ISP between WTau and PTau cells, indicating that Tau phosphorylation mediates an additional effect on ISP. This study provided a potential linkage of abnormal expression and phosphorylation of Tau to the ISP dysfunction in AD.

## Introduction

Alzheimer’s disease (AD) is a common neurodegenerative disease characterized by a progressive decline in both memory and cognitive function. Its pathological features in the brain include amyloid plaques, neurofibrillary tangles (NFTs) formed by hyperphosphorylated Tau and neuronal loss. AD has been strongly associated with metabolic disorders, as patients with type 2 diabetes mellitus (T2D) are at an increased risk of developing it and vice versa, with many shared age-related pathophysiological features like including insulin resistance and disrupted glucose metabolism (Goldstein, 2002; DeFronzo, 2004; Gordon et al., 2018), as well as oxidative and inflammatory stress, amyloid aggregation, neural atrophy, and neurodegeneration (Li and Holscher, 2007; Zhao and Townsend, 2009). Among these dysfunctional brain insulin signaling, termed “brain insulin resistance,” is an important etiological factor in AD (Akhtar and Sah, 2020).

Insulin plays crucial roles in cognition, memory, and neurobehavioral, and regulates the development of the nervous system (Taguchi et al., 2007; Kim and Feldman, 2015; Soto et al., 2019). Peripheral insulin resistance can promote AD onset by increasing the levels of Aβ, Tau phosphorylation, oxidative stress, proinflammatory cytokines, advanced glycation end products, and apoptosis in the brain (Craft, 2005; Sims-Robinson et al., 2010; Cholerton et al., 2011). Moreover, an increasing number of studies have suggested that the brain itself becomes insulin-resistant and mediates or even triggers key pathophysiological events in AD (Rivera et al., 2005; Steen et al., 2005; Salkovic-Petrisic and Hoyer, 2007; Liu et al., 2011; Nuzzo et al., 2015; Rad et al., 2018; Akhtar and Sah, 2020). This is consistent with the observed alterations in many insulin signaling molecules in AD brains (Griffin et al., 2005; Rivera et al., 2005; Steen et al., 2005; Ma et al., 2009; Akhtar and Sah, 2020), and with memory improvements after the selective elevation of forebrain insulin *via* intranasal administration in subjects at high risk for AD (Craft, 2005; Reger et al., 2008). Therefore, AD has been suggested to essentially be “brain diabetes” and is called type 3 diabetes mellitus (Leszek et al., 2017).

Studies have shown that impairment of the insulin signaling pathway (ISP) causally contributes to the pathological mechanisms of Aβ and Tau (Sajan et al., 2016; Rad et al., 2018; Akhtar and Sah, 2020), but it is still unclear if and how Tau affects insulin signaling. A recent study revealed that Tau deletion in mice results in insulin resistance (Marciniak et al., 2017), which led us to explore the effects of increased Tau or phosphorylated Tau on insulin signaling. In addition, it is also expressed in several cancer cells, although its functions in cancer have not yet been addressed (Gargini et al., 2019). Therefore, unveiling the effects of Tau on insulin signaling could have therapeutic implications for both AD and tumors. To address this, we have studied the changes that occur in the levels of some protein levels and their phosphorylation in ISP at basal state, and in the response to insulin stimulation in cells overexpressed Tau and pseudo-phosphorylated Tau.

Lonafarnib, a selective inhibitor of human farnesyltransferase, modulates the functions of Ras superfamily members (O'Meara and Kinsella, 2005) and affects the insulin signaling (Lee et al., 2004; Oh et al., 2006, 2008). It has been used clinically to treat Hutchinson-Gilford progeria syndrome, a disease of premature aging (Ullrich et al., 2013; Gordon et al., 2016, 2018), and can mitigate tauopathy (Hernandez et al., 2019). As AD is an age-related disease, we have tested the effects of lonafarnib on insulin signaling in cells overexpressing Tau with or without pseudo-phosphorylation at AT8 epitopes, and found different effects in these cells.

## Materials and methods

### Antibodies and drugs

Antibodies against the following proteins were used: insulin receptor substrate (IRS), p-IRS (Tyr612) and PTEN (from Abways Technology); PI3K, p-PI3K (p85), p-Akt (Ser473), Akt, p-mTOR (Ser2448), ERK, p-ERK (Thr202/Tyr204), Protein G Magnetic Beads, and LC-3 (Cell Signaling Technology); p-GSK (Ser9), GSK and P62 (Abcam); PP2ACα subunit, PP2ACα with phospho-Y307 (Bioword); demethylated PP2AC subunit (Millipore); Rhes (Signalway Antibody); Flag, T181-Tau, S396-Tau, AT8-Tau, and p-ribosome protein S6 (Ser235/236) (Cell Signaling Technology); p-4EBP1 (T70), p-4EBP1 (T37/46), and 4EBP1 (ABclonal Technology); β-actin was from Santa Cruz Biotechnology. DAPI Alexa Fluor-594 or Alexa Fluor-488 labeled secondary antibodies were from Invitrogen; IRDye labeled secondary antibodies were from LI-COR. Lonafarnib, MK2206, MG132, and bafilomycin A1 were purchased from MCE Company. Thermolysin was from Jinpin Bio Company.

### Cell culture and transfection

A 293 T (human embryonic kidney cell line expressing SV40 T antigen) and SH-SY5Y (human neuroblastoma cell line) cells were obtained from the Cell Bank, Shanghai Institutes for Biological Sciences, Chinese Academy of Sciences (Shanghai, China). The cells were cultured in Dulbecco’s modified Eagle’s medium (DMEM, Gibco) supplemented with 10% fetal bovine serum, 100 U/mL penicillin, and 100 μg/mL streptomycin (Gibco) at 37°C in a humidified atmosphere containing 5% CO_2_ and passaged every 3–4 days.

Although Tau has 86 phosphorylation sites, phosphorylation at the AT8 sites has been shown to be the most pathogenic (Sun and Gamblin, 2009). Therefore, Tau phosphorylated at AT8 for insulin signaling was analyzed in present study. Construction of 2N4R-Tau and Tau with pseudo-phosphorylated at AT8 plasmids were described by our previous report (Cao et al., 2018). AT8 epitope of Tau includes phosphorylation atS199, S202, and T205 (Jeganathan et al., 2008). To mimic Tau phosphorylation at AT8 epitope (PTau), serine and threonine residues at AT8 epitope were substituted to glutamate. To simulate AT8-unphosphorylated Tau (uPTau), serine and threonine residues at AT8 were changed to alanine, which have been described in our previous study (Cao et al., 2018). The cells were transfected with plasmids using Lipofectamine 3000 (Invitrogen) at 24 h after planting (75–80% confluency) as described previously (Cao et al., 2018).

### Immunofluorescent staining

Cells were cultured and maintained on a round slide. After treatment with insulin, cells were washed with 0.01 M PBS followed by a fixation using 4% PFA for 10 min at room temperature. The primary antibody was added to the cells followed by incubation for 48 h at 4°C. Then, the second Alexa Fluor® antibody (Invitrogen) was adopted and incubated with cells for 1 h at 37°C. The nucleic acids were stained with DAPI (Invitrogen). After mounting with anti-fade medium (Sigma), immunofluorescent images were acquired using a fluorescence microscope (Nikon).

### Coimmunoprecipitation

Cells in 100-mm dishes were rinsed with PBS, scraped into 1 mL of lysis buffer containing 50 mM Tris–HCl (pH 7.5), 150 mm NaCl, 1% Nonidet P-40, 0.5% sodium deoxycholate, 1 mM EDTA, and protease inhibitor cocktail (Roche Diagnostics, Mannheim, Germany), which were then sonicated. Cellular debris was removed by centrifugation at 12,000 × g for 15 min at 4°C. The supernatants were incubated with anti-Flag antibody or anti-PTEN for one night at 4°C, then the solution was incubated with Protein G Magnetic Beads for 1 h at room temperature with mixing. The beads were washed with cell lysis buffer four times to remove the unbound immune complex. Then bound proteins were eluted with SDS sample buffer for immunoblot analysis.

### Cellular thermal shift assay and drug affinity responsive target stability assay

The cellular thermal shift assay (CETSA) is used to study thermal stabilization of proteins upon ligand binding in cells (Nagasawa et al., 2020). Samples were prepared from control and drug-exposed cells. For each set, cells were treated with lonafarnib (1 μM) or DMSO as control for 24 h. Cells were washed ice-cold PBS and collected by trypsinization, neutralized by DMEM containing 10% FBS, pelleted at 1000 × g for 5 min. Cells were then incubated in lysis buffer (50 mM Tris–HCl, pH 7.5, 0.5% Triton X-100, 200 mM NaCl, 10% glycerol) containing a complete protease inhibitor cocktail tablet (Roche Diagnostics, Mannheim, Germany) on ice. Cell debris was removed by centrifugation, and the supernatant was aliquoted. Aliquots were heated to designated temperatures for 3 min, and cooled at room temperature for 3 min, and then cooled and centrifuged at 20,000 × g, 4°C for 20 min. The proteins in the supernatant were analyzed by SDS-polyacrylamide gel electrophoresis (SDS-PAGE) and immunoblotting.

The drug affinity responsive target stability (DARTS) assay is based on the principle that the susceptibility of the target protein to proteases is reduced upon drug binding (Lomenick et al., 2009; Pai et al., 2015). In brief, cells transfected different Tau constructs were pretreated with 1 μM of lonafarnib or DMSO as vehicle control for 24 h, washed with pre-cooled PBS, and lysed in 50 mM NaCl, 10 mM CaCl_2_, and 50 mM Tris–HCl (pH7.5), and containing 5% Triton X-100, with protease and phosphatase inhibitor cocktails, and then incubated at 4°C. Lysates were centrifuged for 10 min at 18,000 × g at 4°C to pellet cellular debris. Chilled TNC buffer (50 mM Tris–HCl pH 7.5, 50 mM NaCl, and 10 mM CaCl_2_) was added to the supernatant of protein lysate, and protein concentration of the lysate was measured by the BCA Protein Assay kit (Biomiga, PW0104). Then thermolysin was added to cell lysates at several designated final concentrations to cell lysates. Thermolysin reaction mixtures were incubated at room temperature for 10 min. Samples were boiled immediately after adding loading buffer to stop the digestion, and analyzed by SDS-PAGE and western blotting.

### Western blotting

Cells were collected, washed with ice-cold PBS, lysed in RIPA buffer (150 mM NaCl, 50 mM Tris–HCl, pH7.4, 1% TritonX-100, 1% sodium deoxycholate, and 0.1% each of SDS, sodium orthovanadate, sodium fluoride, and EDTA) with a mixture of protease and phosphatase inhibitors for 30 min on ice. Samples were then sonicated on ice, and the protein concentration in each sample was quantified with a bicinchoninic acid protein assay kit (PW0104, Biomiga). Proteins were resolved by SDS-PAGE and transferred to nitrocellulose membranes, which were then treated with blocking buffer TBST (137 mM NaCl, 0.1% Triton X-100, 20 mM Tris–HCl, pH 7.4) containing 5% BSA for 1 h at room temperature and incubated overnight at 4°C with primary antibody. After being washed with TBST several times, the membranes were reacted with IRDye® secondary antibody for 1 h at room temperature, washed 3 times with TBST, and analyzed with the Odyssey IR Imaging System (Li-COR).

### Quantification and statistical analysis

Analysis was conducted with the GraphPad Prism Version 7.0. For western blotting protein expression data, One-Way ANOVA (with Tukey *Post hoc* test) was performed. The outcomes are presented as bar chart with error bars representing the mean ± SEM, respectively.

## Results

### Effects of wild-type Tau and pseudophosphorylated Tau on the basal protein levels in the insulin signaling pathway

**Figure 1:**
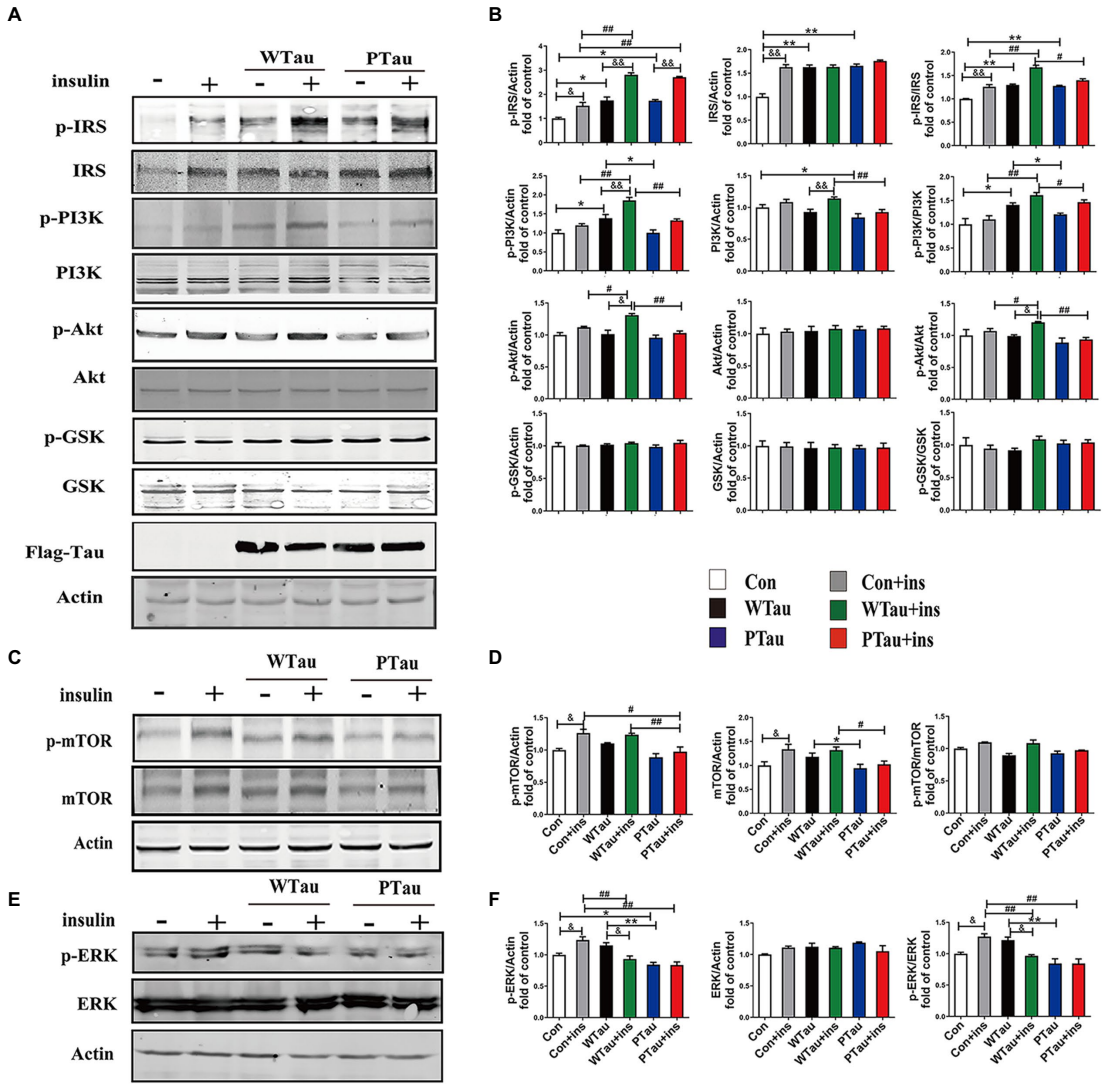
Effects of WTau (Wild-Type Tau) and PTau (AT8E-Tau) overexpressing on the insulin signaling pathway in 293 T cells (insulin treatment for 10 min). **(A,C)** Effects of wild-type Tau and PTau overexpressing on the insulin signaling in 293 T cells with or without insulin treatment. **(B,D)** The densitometric analysis of blots represented in panels **A,C**. **(E)** Effects of wild-type Tau and PTau overexpressing on ERK in 293 T cells with or without insulin treatment. **(F)** The densitometric analysis of blots represented in panel **E**. Data represents the mean ± SEM. *n* = 3. *p < 0.05, **p < 0.01, comparison between groups without insulin treatment; #*p* < 0.05, ##*p* < 0.01, comparison between groups with insulin treatment; &*p* < 0.05, &&*p* < 0.01, comparison between groups with and without insulin treatment.

293 T cells, which lack of Tau (Santa-Maria et al., 2007), were transfected with constructs expressing wild-type 2N4R-Tau (WTau) or Tau with pseudophosphorylation at AT8 epitopes (PTau), and the effects of the overexpression of both on elements in the ISP were investigated by western blotting (Figure 1).

ISP activity is reflected by the phosphorylation of insulin receptor substrate-1 at Tyr612 (p-IRS-1), PI3K at Tyr458 (p-PI3K) and Akt at Ser473 (p-Akt), and the phosphorylation of Akt downstream factors, including GSK3β at Ser9 (p-GSK3β) and mTOR at Ser 2,448 (p-mTOR; Lomenick et al., 2009; Akhtar and Sah, 2020). In this study, a comparison of 293 T cells transfected with WTau and those transfected with an empty plasmid (referred to as control cells) indicated an increased basal ISP activity. Among the ISP proteins, the increases in the levels of p-IRS (0.05), IRS (*p* < 0.01), p-PI3K (p < 0.05), p-IRS/IRS (p < 0.01), and p-PI3K/PI3K (p < 0.05) were statistically significant, whereas p-mTOR and mTOR also showed an increasing tendency (Figures 1A,C).

Compared with the control cells, an increased tendency for ISP elements and their phosphorylation was also observed in PTau cells, and the increases in IRS, p-IRS (Tyr612), p-IRS/IRS were statistically significant (*p* < 0.01, *p* < 0.05, and p < 0.01 respectively). In contrast, compared with the WTau cells, the PTau cells had relatively lower ISP activity; however, only p-PI3K and total mTOR were statistically significant (p < 0.05; Figures 1B,D). Therefore, Tau overexpression could elevate the basal activity of the ISP, whereas Tau phosphorylation at the AT8 epitope somewhat reduced this effect.

### Effects of Tau overexpression on insulin evoked activation of insulin signaling pathway

To investigate the effects of WTau and PTau on the insulin-induced ISP reaction, cells were treated with 200 nM insulin for 10 min (Figure 1). In general, insulin treatment caused ISP activation in the control, WTau, and PTau cells.

The insulin-treated WTau cells were found to have relatively higher levels of p-IRS (Tyr612) (*p* < 0.01), p-PI3K (*p* < 0.01), p-Akt (*p* < 0.05), p-IRS/IRS (*p* < 0.01), and p-PI3K/PI3K (*p* < 0.01), p-Akt/Akt (p < 0.05) as the up-elements of ISP, compared to the insulin-treated control cells, but not downstream factors, such as GSK3β and mTOR (Figures 1A,C). In addition, insulin-treated PTau cells had a significantly higher level of p-IRS (p < 0.01) than the insulin-treated control cells, but not other ISP elements. However, compared to insulin-treated WTau cells, insulin-induced activation of ISP elements tended to be decreased, with decreases in p-PI3K (*p* < 0.01), PI3K (*p* < 0.01), p-Akt (p < 0.01), p-IRS/IRS (p < 0.05), p-PI3K/PI3K (*p* < 0.05), and p-Akt/Akt (*p* < 0.01) levels been significant (Figures 1A,C). These results indicate that the overexpression of Tau could cause a higher reaction in the upstream ISP elements, whereas Tau phosphorylation weakens this effect.

**Figure 2:**
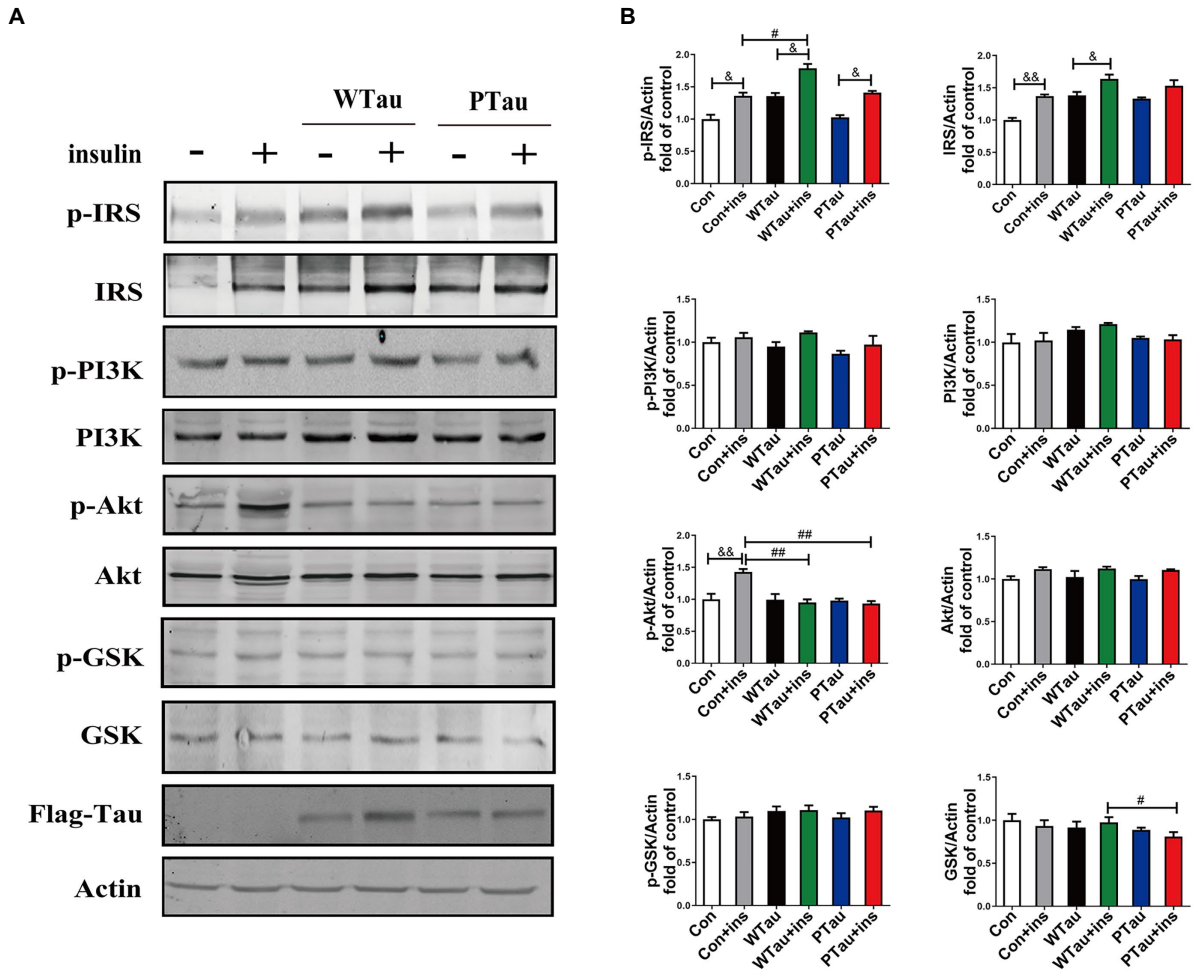
Effects of overexpression WTau (Wild-Type Tau) and PTau (AT8E-Tau) on the insulin signaling pathway in 293 T cells (insulin treatment for 30 min). **(A)** Effects of overexpression wild-type Tau and PTau on the insulin signaling. **(B)** The densitometric analysis of blots represented in panel **A**. Data represents the mean ± SEM. *n* = 3. #*p* < 0.05, ##*p* < 0.01, comparison between groups with insulin treatment; &*p* < 0.05, &&*p* < 0.01, comparison between groups with and without insulin treatment.

To determine whether the extended effects of Tau on the insulin-induced ISP activation, the cells were treated with insulin for 30 min (Figure 2). The upregulation of the upstream portion of ISP elements, p-IRS (*p* < 0.05), IRS (*p* < 0.01), and p-Akt (*p* < 0.01) was still obvious in the control cells (Figures 2A,B). Compared to insulin-treated control cells, insulin-treated WTau cells had significantly higher levels of p-IRS (*p* < 0.05), but not insulin-treated PTau cells. In contrast, insulin-treated WTau and PTau cells showed lower levels of p-Akt (*p* < 0.01) than those in insulin-treated control cells.

**Figure 3:**
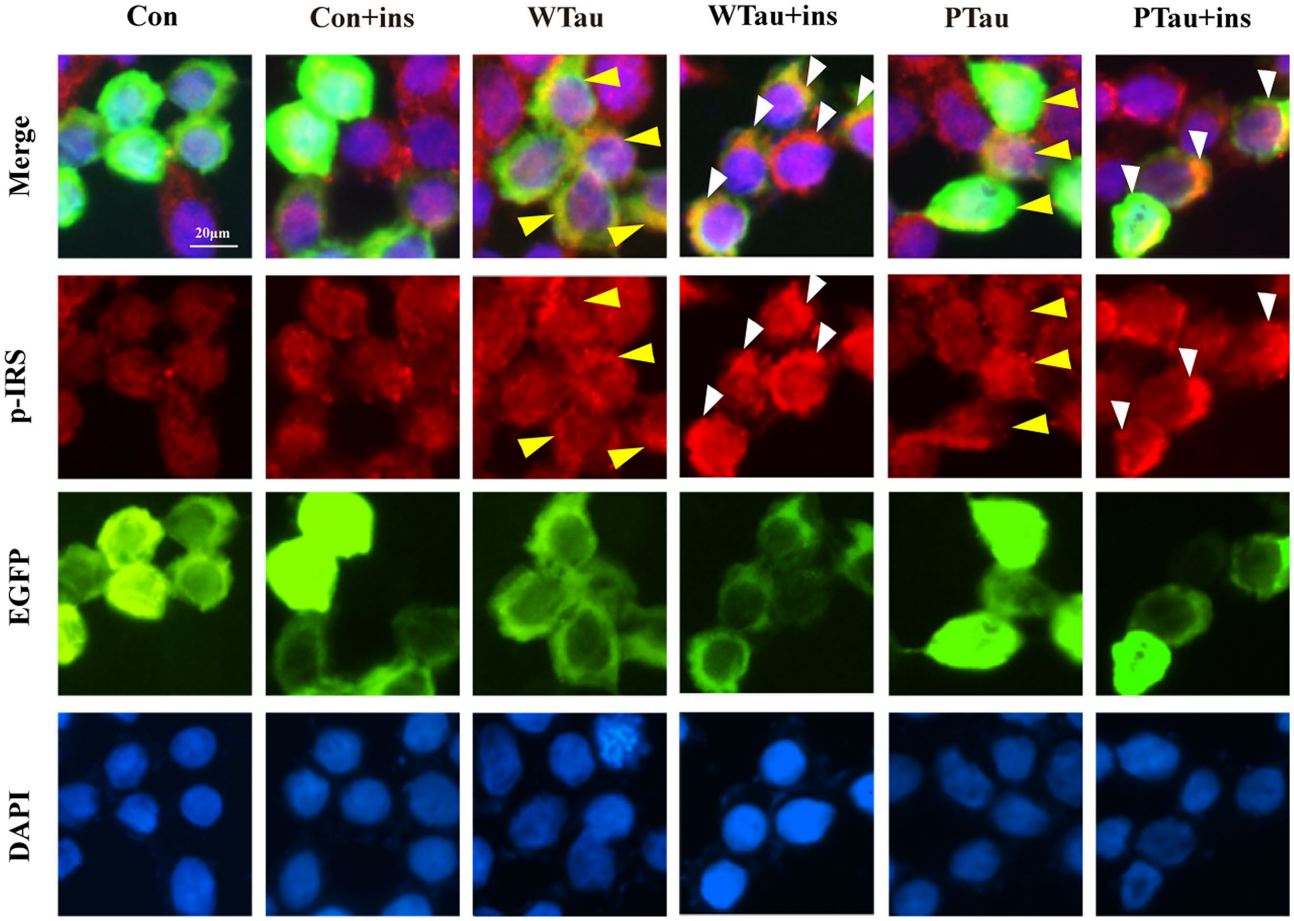
Immunofluorescence staining of p-IRS in 293 T cells with overexpressing wild-type Tau or PTau. 293 T cells were transfected EGFP, wild-Tau (WTau) or Tau with phosphorylated Tau at AT8 residues (PTau), which were fused with EGFP. Then cells were treated with or without insulin (ins) for 10 min. Scale bar = 20 μM. Yellow arrow head indicated the Tau or PTau overexpressed cells without additional insulin treatment. Some of them showed an increase of p-IRS, while others did not. White arrow head indicated an obvious increase of p-IRS in Tau or PTau overexpressing cells treated with insulin.

Meanwhile, immunofluorescence staining also showed that cells overexpressing WTau or PTau contained a higher basal level of p-IRS than that in control cells. After insulin treatment, cells overexpressing WTau or PTau demonstrated more p-IRS distribution than that in insulin-treated control cells. There was no obvious difference of p-IRS staining between the cells overexpressing WTau and PTau (Figure 3). Based on the results of 10- and 30-min insulin treatment, WTau and PTau overexpression affected the insulin-induced signals transduction of ISP.

Besides IRS/PI3K/AKT is the main ISP, insulin is also able to induce Ras and ERK, which could crosstalk with the main pathway, although Ras–ERK appears to be more acitve in response to IGFIR signaling compared to IR signaling (Cai et al., 2017). We observed that the basal level of p-ERK/ERK in WTau cells tended to increase, whereas p-ERK in PTau cells tended to decrease, however both of them did not reach statistical significance. Insulin treatment causes an activation of ERK in control cells (*p* < 0.05), but p-ERK/ERK was declined in WTau cells (p < 0.05), whereas no change in PTau cells (Figures 1E,F). Therefore, insulin induced ERK reaction also altered in WTau or PTau cells.

### Effects of wild-type Tau and pseudo-phosphorylation at AT8 residues on the downstream of mammalian target of rapamycin

The alteration of mTOR signaling, especially for factors downstream of mTOR, is known to occur early in the progression of AD (Tramutola et al., 2015) and at the severe stages of AD (Sun et al., 2014). Studies have suggested that Aβ is involved in abnormalities in the PI3K/Akt/mTOR axis in AD (Caccamo et al., 2010; Gupta and Dey, 2012; O'Neill et al., 2012). This study evaluated the effects of the overexpression of Tau and its pseudo-phosphorylated form on the phosphorylation of ribosomal protein S6 and eIF4E binding protein (4EBP), the downstream factors of mTOR, which are reportedly elevated in AD (Tramutola et al., 2015; Sun et al., 2020).

**Figure 4:**
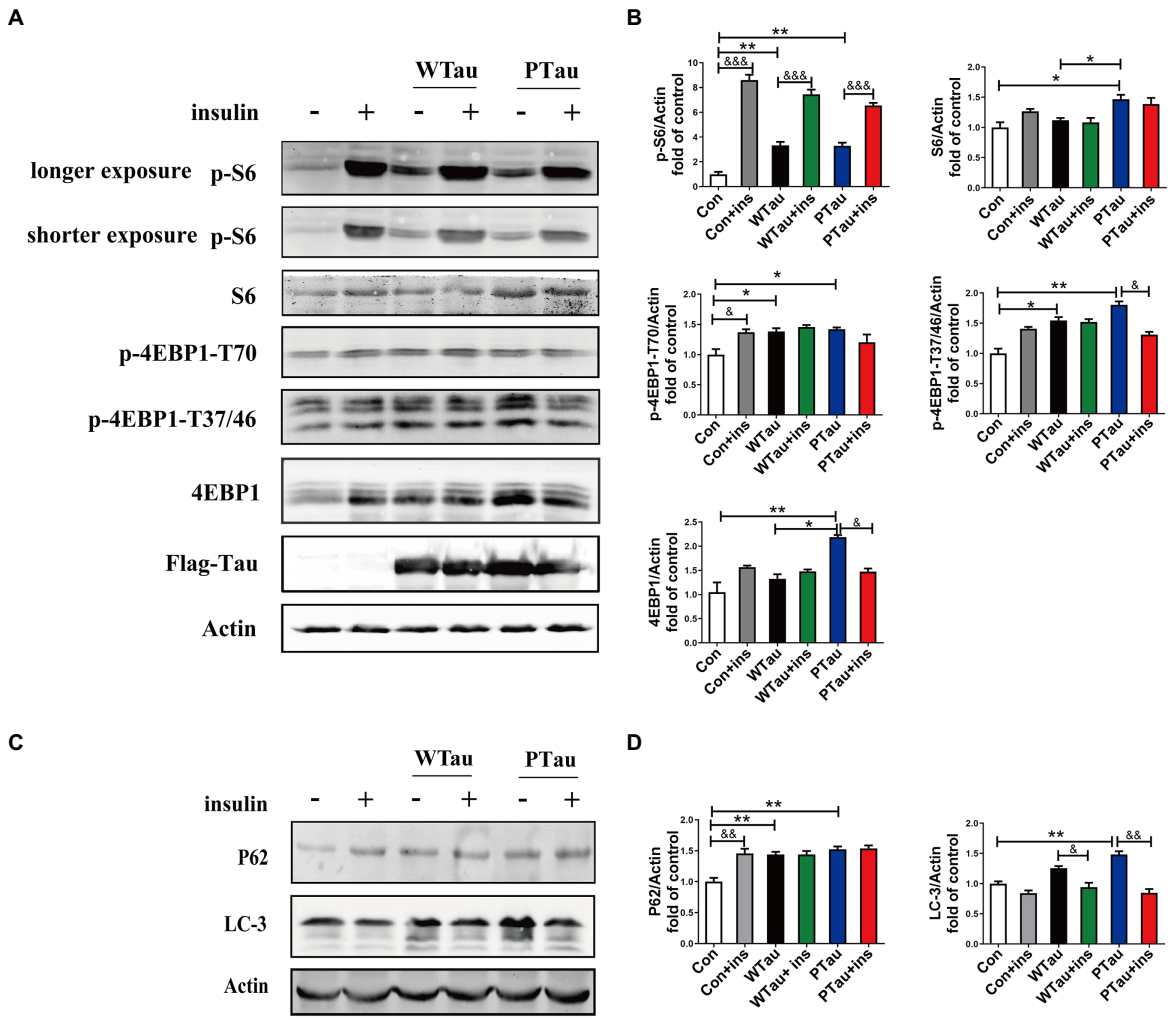
Effects of WTau (Wild-Type Tau) and PTau (AT8E-Tau) overexpressing on the downstream of insulin signaling pathway in 293 T cells. **(A)** Effects of WTau and PTau overexpressing on the levels of S6, p-S6, 4EBP1, and p-4EBP1 with and without insulin (Ins) treatment for 10 min. **(B)** Densitometric analysis of blots represented in panel **A**. **(C)** Effects of WTau and PTau overexpressing on the levels of P62 and LC-3 that are involved in autophagy in 293 T cells. **(D)** Densitometric analysis of blots represented in panel **E**. Data represents the mean ± SEM. *n* = 3. *p < 0.05, **p < 0.01, comparison between groups without insulin treatment; &*p* < 0.05, &&*p* < 0.01, &&&*p* < 0.001, comparison between groups with and without insulin treatment.

Compared with the control cells, the basal levels of p-S6 (3.31 fold, *p* < 0.01), p-4EBP1-T70 (1.85 fold, p < 0.05), and p-4EBP1-T37/46 (1.54 fold, *p* < 0.05) in WTau cells were significantly increased, and the increase in phosphorylation of S6 was the most dramatic (Figure 4A). In addition, PTau cells showed an increased levels of p-S6 (3.3 fold, *p* < 0.01), p-4EBP1-T70 (1.94 fold, p < 0.05) and p-4EBP1-T37/46 (1.7 fold, p < 0.01). There were no significant differences in these proteins between the WTau and PTau cells.

However, WTau and PTau overexpression significantly suppressed the insulin-induced increase in p-S6 (2.25 and 1.99 fold, respectively), in contrast to that of control cells (8.6 fold; Figure 4A). For p-4EBP1-T70, insulin treatment caused an increase for control cells (*p* < 0.05), but did not change WTau cells, and even caused a reduction of PTau cells. In addition, insulin-induced 4EBP1 phosphorylation at the T37/46 sites in these cells showed a pattern similar to that of p-4EBP1-T70. Therefore, these results indicated that the overexpression of Tau and its phosphorylated form could significantly elevate the basal levels of pS6 and p-4EBP1, but alleviate the insulin-induced increase in p-S6 and p-4EBP1, particularly in PTau cells.

**Figure 5:**
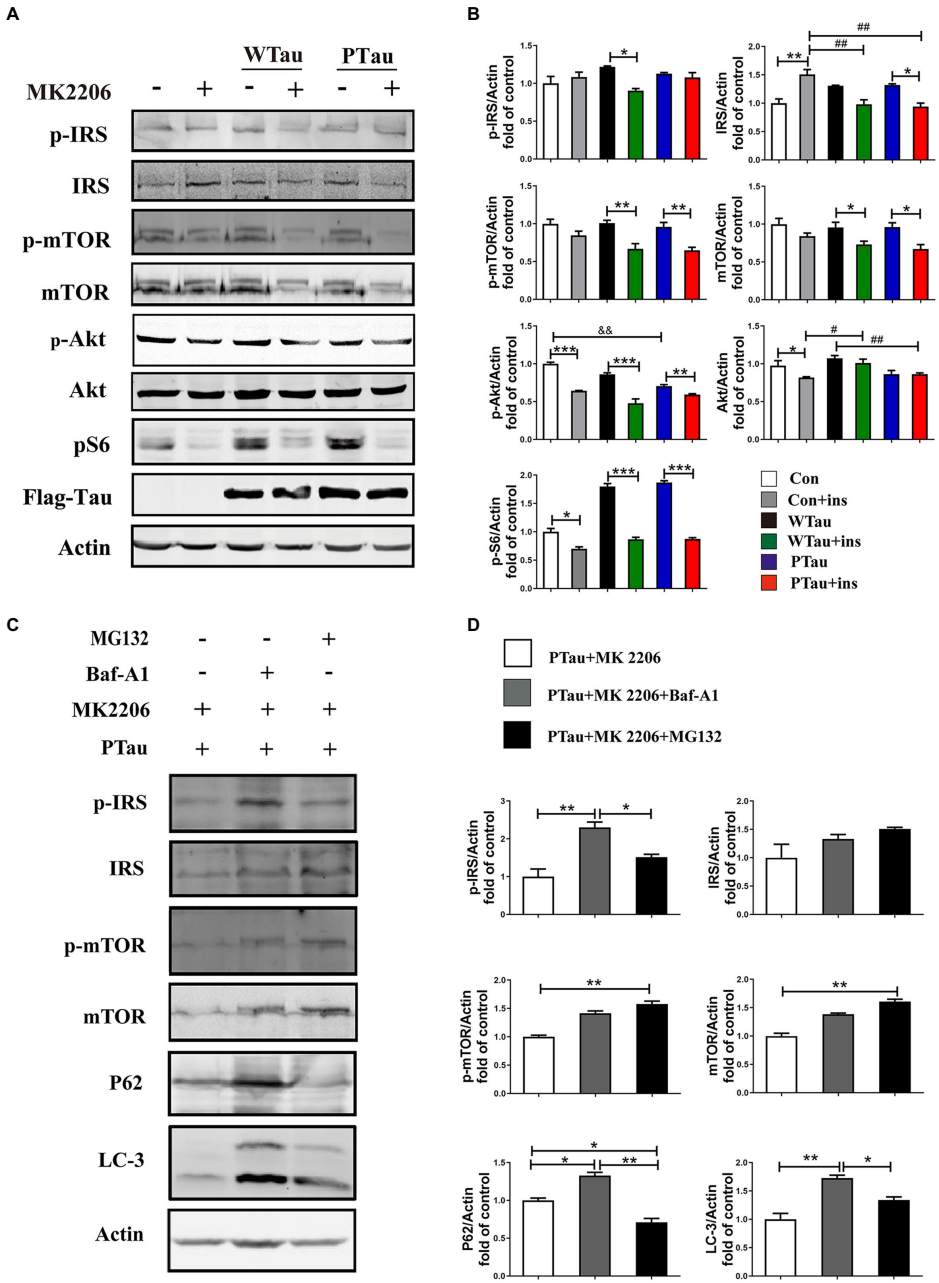
The effects of Akt inhibition on the insulin signaling in 293 T cells overexpression of WTau or PTau. **(A)** Akt inhibition by MK 2206 (5 μM, 12 h) treatment suppressed insulin signaling system in 293 T cells overexpression of Wild-Type Tau (WTau) or Tau with pseudo-phosphorylation at AT8 residues (PTau). **(B)** Densitometric analysis of blots represented in panel **A**. Data are mean ± SEM. *n* = 3. *p < 0.05, **p < 0.01, ***p < 0.001, comparison between groups with and without insulin treatment; #*p* < 0.05, ##*p* < 0.01, comparison between groups with insulin treatment; &&*p* < 0.01, comparison between groups without insulin treatment. **(C)** Autophagy inhibitor (Baf-A1, 5 nM) or proteasome inhibitor (MG132, 5 μM) augmented the IRS, IRS-P, mTOR, and p-mTOR levels in PTau-293 T cells treated with the Akt inhibitor MK2206. **(D)** Densitometric analysis of blots represented in panel **C**. Data represents the mean ± SEM, *n* = 3. *p < 0.05, **p < 0.01.

In this study, the overexpression of WTau and PTau in 293 T cells significantly affected the basal levels of the upstream factor p-IRS and downstream factors such as p-S6 and p-4EBP1, whereas the phosphorylation of Akt, a midstream factor, was barely affected. Akt is known to be an important connecting link between the up- and the down-stream elements in ISP (Copps and White, 2012). To explore whether Akt contributes to the abnormalities in these upstream and downstream factors in WTau and PTau cells, the cells were treated with MK2206, an Akt inhibitor (Hirai et al., 2010; Xiang et al., 2017; Bjune et al., 2018). After MK2206 treatment for 12 h, the decline of p-Akt was expected. Moreover, the levels of both p-mTOR and mTOR in WTau- and PTau-transfected cells were significantly downregulated (*p* < 0.01 and *p* < 0.05, respectively); however, the decline was not significant in the control cells (Figures 5A,B). Moreover, the MK2206 treatment caused a decline in p-S6 to a similar level among these cells (Figures 5A,B). Therefore, we speculated that Akt is involved in ISP dysregulation in WTau and PTau cells.

Furthermore, MK2206 upregulated IRS levels in the control cells (p < 0.01), but tended to downregulate IRS levels in WTau and PTau cells (*p* < 0.05; Figures 5A,B). These results indicate that the regulation of insulin signaling in WTau- or PTau-transfected cells was different from that in control cells. Blockage of either autophagic degradation with bafilomycin A1 (Baf-A1) or proteasome degradation with MG132 in MK2206 treated PTau cells (Figures 5C,D) elevated the levels of mTOR and IRS, suggesting that Akt plays a role in the regulation of mTOR and IRS levels in Tau overexpressing cells.

### Effects of wild-type Tau and pseudo-phosphorylation at AT8 residues on insulin-regulated autophagy

The autophagic system is another downstream effector of the mTOR pathway. The effects of WTau and PTau on autophagy were then investigated by examining SQSTM1P62 and LC-3 (microtubule-associated protein 1 light chain 3)-II (Figures 4C,D). The results showed that WTau and PTau cells had higher basal levels of P62 than the control cells (*p* < 0.01); while the basal level of LC-3 in WTau and PTau cells also tended to be higher than that in the control cells, especially for PTau cells (p < 0.01). Therefore, the overexpression of WTau or PTau affects the basal condition of the autophagic system. However, insulin treatment for 30 min did not cause an increase in P62 in WTau- or PTau -cells as control cells, whereas it downregulated the LC-3 levels in WTau and PTau cells (*p* < 0.05 and *p* < 0.01, respectively) to a level close to that of the insulin-treated control cells.

### Effects of wild-type Tau and pseudo-phosphorylation at AT8 residues overexpression on protein phosphatase

The ISP is regulated by phosphatases, among which PP2A (protein phosphatase 2A) and PTEN (phosphatase and tensin homolog) play critical roles (Jafari et al., 2014; Marciniak et al., 2017; Li et al., 2020).

**Figure 6:**
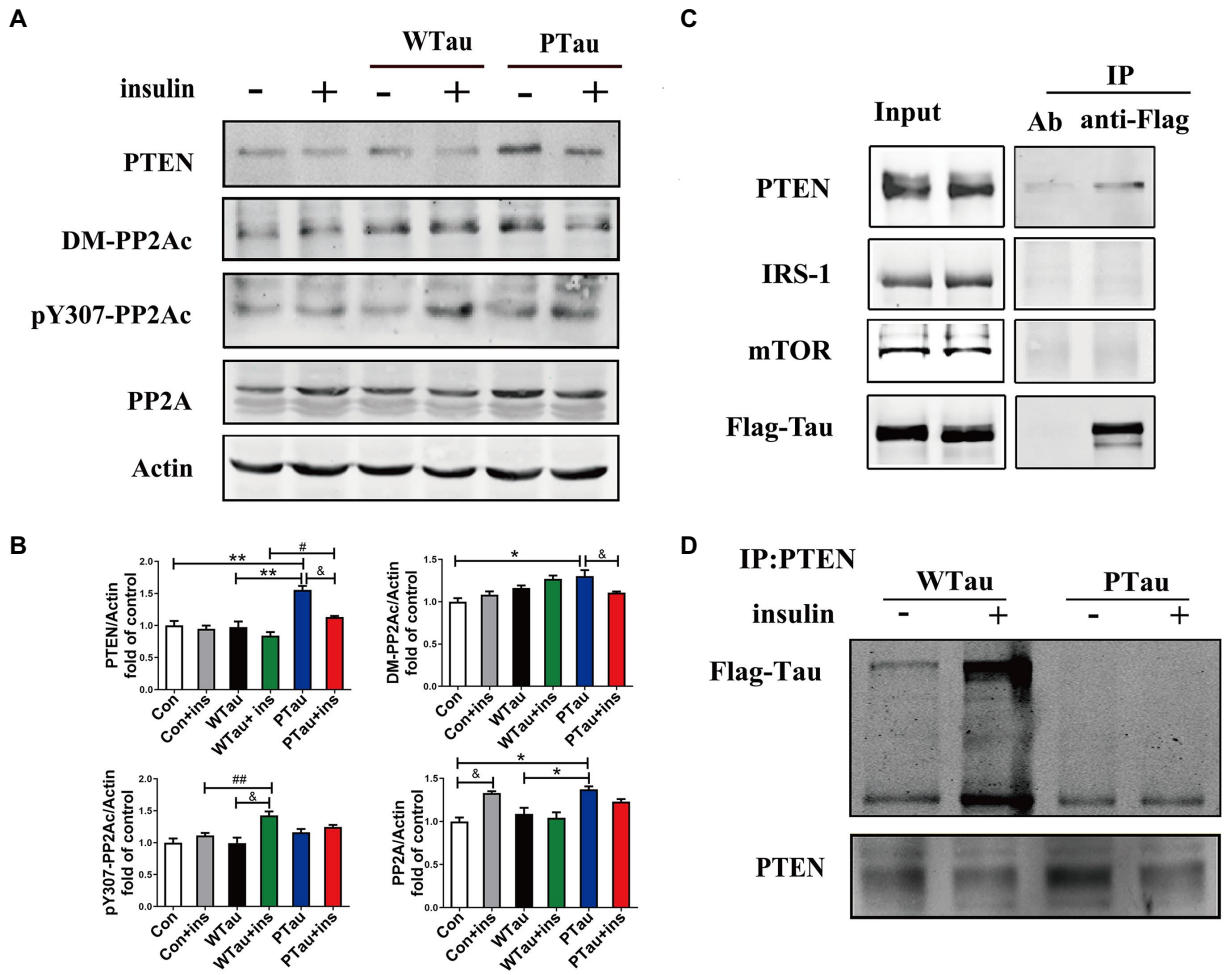
Effects of overexpression of WTau and PTau on protein phosphatase. **(A)** Effects of overexpression WTau and PTau on the levels of PTEN and PP2A. PP2Ac: PP2A catalytic subunit. pY307-PP2Ac: PP2Ac phosphorylated at Tyr307. DM-PP2Ac: PP2Ac de-methylated at Leu309. **(B)** Densitometric analysis of blots represented in panel **A**. Data represents the mean ± SEM. *n* = 3. *p < 0.05, **p < 0.01, comparison between groups without insulin treatment; &*p* < 0.05, comparison between groups with and without insulin treatment; #*p* < 0.05, ##*p* < 0.01, comparison between groups with insulin treatment. **(C)** Co-immunoprecipitation experiments analyzed the interaction between wild-type Tau (WTau) and different insulin-signaling members in cells transfected with EGFP-wild-type 2N4RTau-Flag, showing an association between Tau and PTEN. Immunoprecipitation was performed with unimmunized animal antibody (Ab) and anti-Flag tag antibody, the immunoprecipitates were analyzed by PTEN, IRS-1, and mTOR. **(D)** The different binding of WTau and PTau (EGFP-wild-type 2N4RTau-Flag with pseudo-phosphorylation at AT8 residues) to PTEN. Immunoprecipitation was performed with anti-PTEN antibody, the immunoprecipitates were analyzed by anti-Flag antibody.

Results also showed that the basal level of the catalytic subunit of PP2A (PP2Ac) was increased in PTau cells but not in WTau cells relative to that in the control cells (Figures 6A,B). Demethylation at Leu309 (DM-PP2Ac) or phosphorylation at Tyr307 (p-PP2Ac) would reduce the activity of PP2Ac (Wang et al., 2015; Javadpour et al., 2019). The basal level of DM-PP2Ac tended to increase in WTau cells, but significantly increased in PTau cells (*p* < 0.05). Insulin treatment induced a slight increase of DM-PP2Ac in WTau cells however, it decreased in PTau cells (*p* < 0.05).

In addition, WTau or PTau transfection did not significantly affect the basal levels of pY307-PP2Ac. Insulin treatment only caused a significant increase in pY307-PP2Ac in the WTau cells (p < 0.05), but not in PTau or control cells. Based on these results, WTau and PTau overexpression may differentially affect PP2A, which may partially devote to the effects of Tau and its phosphorylation state on ISP.

There was no obvious change in PTEN in the WTau cells, but an increase in PTau cells (1.6 fold, *p* < 0.01), relative to that of control cells was observed. Treatment with insulin for 30 min resulted in a decrease tendency in PTEN in all these cells, among which the decline in PTau cells was significant (*p* < 0.05; Figures 6A,B). Besides, this study demonstrated that WTau could co-immunoprecipitate (CoIP) with PTEN, but very little with IRS1 and mTOR (Figure 6C). Furthermore, CoIP with the PTEN antibody also showed the binding between PTEN and WTau (Figure 6D). Thereafter, we evaluated the binding between PTEN and PTau, which showed that this binding was very weak (Figure 6D). Therefore, Tau phosphorylation may compromise the function of Tau in regulating PTEN function activity.

### Effects of lonafarnib on the insulin signaling pathway in SH-SY5Y cells expressing wild-type Tau or pseudo-phosphorylation at AT8 residues

**Figure 7:**
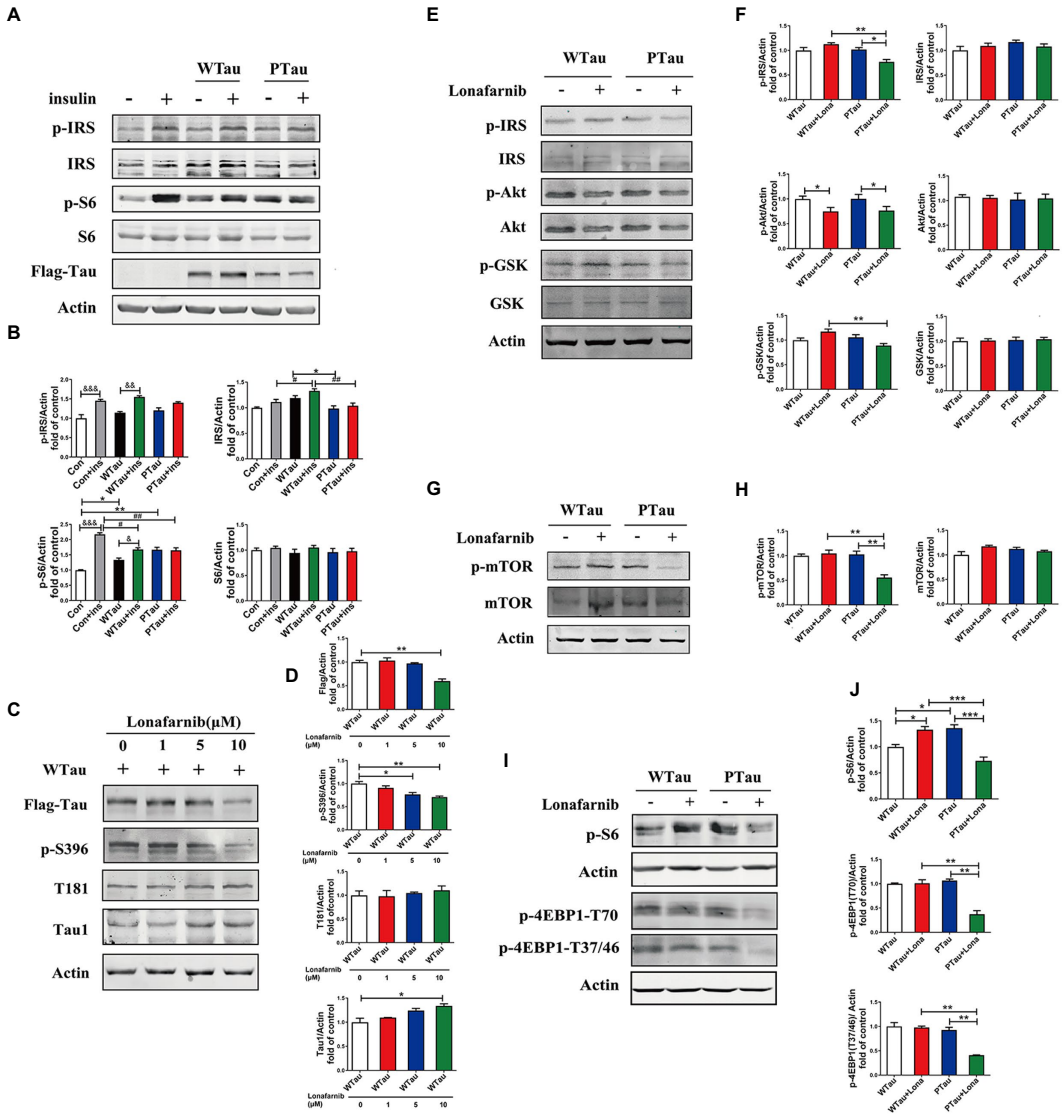
Differential effects of lonafarnib on insulin signaling system in SH-SY5Y cells overexpressing WTau and PTau. **(A)** Effects of overexpressing Wild-Type Tau (WTau) and AT8E-Tau (PTau) on the insulin signaling system with or without insulin treatment for 10 min in SH-SY5Y cells. **(B)** Densitometric analysis of blots represented in panel **A**. Data represents the mean ± SEM. *n* = 3. *p < 0.05, **p < 0.01, comparison between groups without insulin treatment; &*p* < 0.05, &&*p* < 0.01, &&&*p* < 0.001, comparison between groups with and without insulin treatment; #*p* < 0.05, ##*p* < 0.01, comparison between groups with insulin treatment. **(C)** Effects of lonafarnib on the phosphorylation of Tau in cells transfected with WTau. The phosphorylation at pS396 and pT181 of Tau were detected by p-S396 and T181 antibodies respectively, and Tau with dephosphorylated serine sites at 195, 198, 199, and 202 were detected by Tau1 antibody. The transfected WTau level was analyzed by anti-Flag tag antibody. **(D)** Densitometric analysis of blots represented in panel **C**. Effects of lonafarnib (1 μM, 24 h) on the insulin signaling upstream factors of pathway including IRS, PI3K, Akt, and GSK **(E)**, mTOR **(G)** and its downstream factors S6 and 4EBP1 **(I)** in WTau and PTau transfected SH-SY5Y cells. **(F,H,J)** Densitometric analysis of blots represented in panels **E,G,I**. Data represents the mean ± SEM, *n* = 3. *p < 0.05, **p < 0.01, ***p < 0.001.

In contrast the effects of Tau on ISP on 293 T cells, the non-neuronal cells, as described in the above results, WTau or PTau overexpressing in the human neuroblastoma cell line SH-SY5Y cells also showed deregulation of ISP, with a slight increasing tendency of p-IRS the upstream factor of ISP, but a significant increase in p-S6 (*p* < 0.05) in basal conditions (Figures 7A,B). In addition, insulin-induced S6 phosphorylation was also suppressed in WTau or PTau overexpressed SH-SY5Y cells (Figures 7A,B), as seen in 293 T cells (Figure 1).

Previous studies have shown that the farnesyltransferase (FTase) inhibitor lonafarnib regulates ISP (Lee et al., 2004; Oh et al., 2006, 2008). Therefore, we evaluated whether it affected ISP in SH-SY5Y cells overexpressing WTau or PTau (Figure 7). SH-SY5Y cells overexpressing WTau were treated with various concentrations (1, 5, and 10 μM) of lonafarnib for 24 h (Figures 7C,D). The downregulation of Tau level and Tau phosphorylation at S396 occured after the lonafarnib treatment at 5 μM, and was significant with 10 μM lonafarnib (*p* < 0.01). However, phosphorylation at T181 was not affected. The signals of the Tau unphosphorylated epitope recognized by the Tau1 antibody tended to increase after treatment with 5 and 10 μM of lonafarnib. Thereafter, 1 μM lonafarnib was used to analyze ISP to minimize the effects of lonafarnib on Tau phosphorylation.

WTau cells treated with 1 μM lonafarnib for 24 h showed an increase in the phosphorylation of ISP elements, among which the increase in p-S6 was significant (p < 0.05; Figure 7I), but the level of p-Akt was decreased (p < 0.05; Figure 7E). In contrast, treatment of PTau cells with lonafarnib resulted in a decrease in ISP activity, appearing the downregulation of p-IRS (*p* < 0.05), p-Akt (p < 0.05; Figures 7E,F), p-mTOR (*p* < 0.01; Figures 7G,H), and the downstream factors of mTOR including decreased levels of p-S6 (*p* < 0.001), p-4EBP1-T70 (*p* < 0.01), and p-4EBP1-T37/46 (*p* < 0.01; Figures 7I,J). Therefore, the phosphorylation of Tau at the AT8 epitope could modulate the effect of lonafarnib on the ISP.

### Analysis of Rhes with cellular thermal shift assay and drug affinity responsive target stability assays

Rhes (Ras homolog enriched in striatum), one of FTase substrates, in known to regulate Akt (Bang et al., 2012; Harrison et al., 2013). We then investigated whether Tau phosphorylation could affect the possible action of lonafarnib on Rhes using methods of DARTS and CETSA (Lomenick et al., 2009; Jafari et al., 2014; Park et al., 2016).

**Figure 8:**
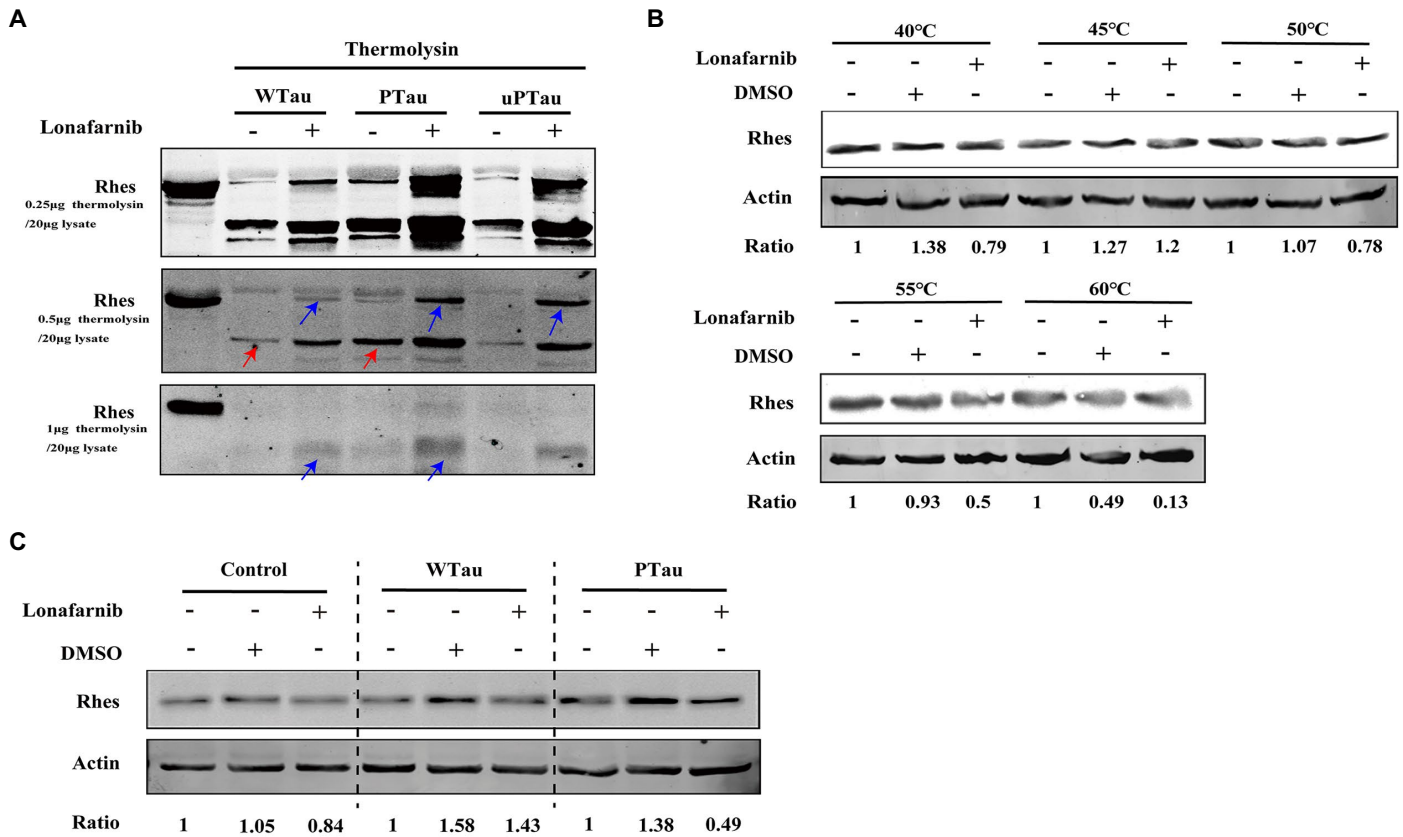
Rhes phosphorylation modulates the effect of lonafarnib on Rhes in SH-SY5Y cells. **(A)** Rhes from cells transfected with Wild-type Tau (WTau), Tau with pseudo-phosphorylation at AT8 residues (PTau) and pseudo-nonphosphorylated at (uPTau) with or without lonafarnib treatment were analyzed by DARTS assay. Results showed that lonafarnib treatment caused an increase of resistance for thermolysin digestion, and Rhes in AT8E-Tau expressing cells appeared more resistance for thermolysin than that in WTau cells. Red arrows indicated the fragment in samples without lonafarnib treatment. Blue arrows indicated the Rhes immunopositive bands in samples with lonafarnib treamtent. **(B)** The effects of lonafarnib on the thermostability of Rhes at 40–60°C was measured by CETSA. **(C)** The effects of lonafarnib on the Rhes thermostability in the lysates from control cells were analyzed by CETSA, WTau and AT8E-Tau cells at 55°C. Ratio refers to the relative Ratio of Rhes/Actin.

In DARTS assay, SY5Y cells over expressing WTau, PTau, and uPTau (pseudo-non-phosphorylated at AT8 epitope) were treated with or without the lonafarnib, lysis of SY5Y cells were then digested with 0.25, 0.5 or 1 μg thermolysin/20 μg lysate (Figure 8A). The results showed that Rhes in PTau cells was more protective from thermolysin digestion than that in WTau cells. Lonafarnib treatment caused more intact or fragmented Rhes to be left after thermolysin (Figure 8A). This effect of lonafarnib was more obvious in PTau cells comparing that in WTau or uPTau cells. Therefore, lonafarnib affected the state of Rhes more efficiently in cells expressing PTau than that of WTau, which may be related to the different effects on the ISP for these cells.

With the CETSA assay (Figure 8B), lonafarnib treatment of control cells did not cause an increase in Rhes in the soluble fraction but even appeared to decrease at 55°C (Figure 8B). Moreover, the lonafarnib treatment also resulted in a reduction in Rhes in the soluble fraction in both WTau and AT8E-Tau SH-SY5Y cells (Figure 8C). Typically, small-molecule drugs increase the heat stability of their target proteins (Jafari et al., 2014; Friman, 2020). Therefore, these results do not provide sufficient evidence for the direct binding between lonafarnib and Rhes. However, the DARTS results suggest that lonafarnib could affect the biophysical chemical state of Rhes, in which Tau phosphorylation plays a regulatory role.

## Discussion

Epidemiological studies have focused on the relationship between AD and the most common disorder of the insulin system, namely, “diabetes mellitus (DM)” (Goldstein, 2002; Biessels et al., 2006; Ghasemi et al., 2013; Dolan et al., 2020; Friman, 2020) and brain insulin resistance is considered a risk factor for AD and related neurodegenerative disorders (Rivera et al., 2005; Steen et al., 2005; Salkovic-Petrisic and Hoyer, 2007; Liu et al., 2011; Nuzzo et al., 2015; Rad et al., 2018; Akhtar and Sah, 2020; Kellar and Craft, 2020). Insulin resistance is defined as a dysfunction in the ability of target tissues to mount a normal response to insulin. Several studies have suggested that Aβ takes a role in insulin resistance of brain. However, the mechanisms underlying the insulin-resistant state of the brain in AD are poorly understood. In present study, we evaluated the effects of Tau overexpression or pseudo-phosphorylated Tau on insulin signaling.

The upstream factors of the ISP include the IRS/PI3K/Akt signaling pathway, in concert with mTOR, which affects the phosphorylation of downstream elements S6 and 4EBP1. The analysis of those factors showed that transfection with WTau or PTau upregulated the resting/basal activities of some ISP elements, especially for the upward elements and the factors downstream of mTOR. However, we noted that the increase in the magnitude of insulin-stimulated phosphorylation/activity of insulin-signaling components tended to decline, particularly the downstream factors of mTOR, such as S6 and 4EBP1, in WTau and PTau cells. This blunt reaction was more obvious in PTau cells. Interestingly, the brains of insulin-resistant mice and monkeys presented pathological changes in Tau also elevated the basal activity of ISP elements with little or no additive response induced by insulin treatment (Sajan et al., 2016). Therefore, the pathological changes in Tau may upregulate the tonic activity of ISP while suppressing its phasic activation of ISP stimulated by insulin. In present study, we used HEK 293 T cells because they are non-neuronal cells lacking Tau expression (Santa-Maria et al., 2007), and the clear background of Tau might lead a stronger reaction after Tau overexpression. Results really showed that the alterations of the ISP caused by overexpression of Tau or pseudo-phosphorylated Tau is more obvious in 293 T cells than those in SH-SY5Y cells. The relative lower alterations of IPS in SH-SY5Y cells might be due to the background expression of different Tau isoforms.

ISP signaling *via* mTOR controls autophagy. The basal levels of SQSTM1/P62 and LC-3 both increased in WTau and PTau cells, suggesting that Tau accumulation represses autophagy flux (Feng et al., 2020). Although insulin treatment led to a decrease of LC-3 in both control and Tau transfected cells, it caused an increase in P62 in control cells but not in WTau and PTau cells, suggesting that Tau modifies the effects of insulin on autophagic system. In addition, mTOR promotes protein synthesis, cellular growth and proliferation by enhancing important translational components, such as 4EBP1 and ribosomal protein S6. The function of eIF4E in cap-dependent translation could be blocked by 4EBP1 which can be suppressed by phosphorylation, whereas the phosphorylation of S6 regulates translation initiation (Proud, 2005; Ruvinsky et al., 2006; Caccamo et al., 2010; Tramutola et al., 2015; Mueed et al., 2018). An increase in p-4EBP1 and p-S6 has been found to be positively correlated with Tau phosphorylation in AD (Li et al., 2005). The maladjustment of this signaling by Tau overexpressing and its phosphorylated form may be important because this static hyper-ISP leads to or is accompanied by the impairment of physiological fluctuations in neuronal functions, which may directly contribute to AD pathogenesis (Caccamo et al., 2010; Tramutola et al., 2015; Mueed et al., 2018).

The presence of basal activity of insulin signaling might be not only due to the constitutive ligand independent activity of insulin signaling (Frazier et al., 2020), but also the actions by other signal molecules (Milstein and Ferris, 2021). Several Gluts (glucose transporter) have been found in neurons (Koepsell, 2020). Constitutive activation of insulin receptor in hippocampal neurons increases the expression of Glut3 and upregulates glucose uptake and metabolism, and the Glut3 is supposed to serve housekeeping uptake of glucose into neurons (Frazier et al., 2020). Considering Gluts could take a role in glucose sensing (Frazier et al., 2020), glucose uptake might be possibly involved in regulation of constitutive insulin signaling. In contrast, insulin evoked signaling might perform a more delicate function. Although insulin could transfer into brain, evidence indicated that insulin is synthesized by subpopulations of cortical and hippocampal neurons. Brain-derived insulin is supposed to provide local stimuli for rapid upregulation of Glut4, an insulin-sensitive glucose transporter in neurons with high energy demand (Koepsell, 2020), which is involved in memory acquisition (Pearson-Leary and McNay, 2016). Furthermore, insulin in brain modulates neurite outgrowth and dendritic spine formation, regulates expression of neurotransmitter receptors and activity-dependent synaptic plasticity. Insulin has a crucial role in development and maintenance of excitatory synapses, and neuronal survival. Therefore, the deregulated reaction tune of insulin stimulated signaling caused by overexpression of Tau or its phosphorylated form would affect brain functions.

The mechanism of Tau in ISP remains unknown. Akt acts as a link between the upstream and downstream ISP (Copps and White, 2012). Our results showed that the Akt inhibitor MK2206 downregulated the levels of p-S6 (Figure 5A) in WTau and PTau cells, to the level in control cells. Therefore, dysregulation of downstream ISP in WTau and PTau cells was mediated by an upward stream of the ISP axis.

Moreover, in present study, MK2206 could downregulate mTOR and IRS levels *via* degradation mechanisms, which was more obvious in WTau and PTau cells relative to control cells. The differences in regulation of IRS level between control cells and Tau cells, may be related to the changes in the autophagic system in Tau overexpressing cells, appearing the increase of LC-3 and P62 (Figure 4C). Therefore, these cells might compensatively increase the ability of autophagy attempting to eliminate the overexpressed Tau. The activation of autophagy induced by MK2206 (Pantazi et al., 2022) in those cells would effectively evoke the degradation of some proteins including IRS. However, the detailed mechanism still remained to be explored. Therefore, MK2206, a well-tolerated and safe Akt blocker (Xiang et al., 2017) might be potentially used to rectify ISP in AD.

It has been knows that insulin induced ERK activation could enhanced the activity of mTOR (Mendoza et al., 2011). The lack or suppressive response of ERK phosphorylation under insulin treatment might be the partial reason for the blunt of S6 response to insulin in WTau and PTau cells. Therefore, ERK might also take a role in mediating the malfunction of insulin signaling.

For phosphatase PTEN, the negative regulator of upstream ISP (Gupta and Dey, 2012; Marciniak et al., 2017; Li et al., 2020), the results demonstrated the binding between WTau and PTEN, implying a blockage of PTEN (Marciniak et al., 2017), neverthless, the binding between PTau and PTEN was significantly reduced. Considering the limited difference of upstream ISP dysfunction between WTau and PTau cells, we supposed that other mechanisms may be involved in the ISP in PTau cells. In addition, the phosphatase PP2A is also involved in the signaling transmission of insulin (Javadpour et al., 2019). Our results showed that the level of PP2Acα, the catalytic α subunit, was increased in PTau cells but not in WTau overexpressing cells. The demethylation of this subunit (DM-PP2Ac) or phosphorylation of the Y307 site (pY307-PP2Ac) reflects a decline index in activity (Wang et al., 2015; Javadpour et al., 2019). In this study, the basal level of DM-PP2Ac tended to increase in both WTau and PTau cells, and was significant for PTau cells. Insulin treatment caused a down-regulation of DM-PP2Ac in PTau cells and upregulated pY307-PP2Ac in WTau cells. Therefore, deregulation of PP2A activity may be invloved in overexpressing WTau or PTau induced the dysfunction of ISP signaling.

Lonafarnib is a farnesylation inhibitor that has been used to treat cancers (Lee et al., 2004; Oh et al., 2006) and premature aging disease (Ullrich et al., 2013; Gordon et al., 2016, 2018). A recent study also found that lonafarnib suppresses tauopathy in the rTg4510 mice (Hernandez et al., 2019). Our results showed that lonafarnib downregulated ISP activity in PTau cells, evidenced by the decrease in the levels of p-IRS, p-Akt, p-mTOR, p-S6, and p-4EBP1. In contrast, lonafarnib barely affected the ISP of WTau cells. These results inferred that Tau phosphorylation could regulate the effects of lonafarnib on the ISP.

Lonafarnib appeares to have the opposite effect on the ISP in different cancer cell lines (Oh et al., 2008). Tau is expressed in several cancer cells (Gargini et al., 2019), and the different actions of lonafarnib in WTau and PTau cells observed in the present study suggested that the evaluation of Tau and its phosphorylation might be valuable for the development of cancer treatments. Many substrates of lonafarnib have been identified among which Rhes participates in tauopathy (Hernandez et al., 2019; Ehrenberg et al., 2021) and is involved in ISP signaling (Bang et al., 2012; Harrison et al., 2013). The analysis with DARTS and CETSA methods (Lomenick et al., 2009; Jafari et al., 2014; Park et al., 2016), indicate that lonafarnib could affect the state of Rhes, possibly by changing the spatial structure or interacting with other molecules. Moreover, Rhes in lonafarnib-treated PTau cells showed a significantly higher resistance to thermolysin than that in WTau cells, which may be attributed to the different effects of lonafarnib on the ISP in WTau- or PTau-expressing cells. It should be noted that, in the present study, we utilized 2N4R-Tau, the effects of other alternatively spliced form of Tau on the ISP will required further investigation.

In brief, our results demonstrate that overexpression of Tau and Tau with pseudo-phosphorylation at AT8 residues causes the upregulation of basal/tonic ISP, and a suppression of insulin induced the phasic activation of ISP, which may be important in the impairment of physiological fluctuations of neuronal functions in AD pathology. This dysregulation of insulin evoked signaling transmission was more obvious in PTau cells. Moreover, we found the different effects of lonafarnib on ISP in WTau and PTau cells, which may be related to that Rhes deferentially affected. These results will aid in our understanding of the influence of Tau and its phosphorylation on insulin signaling.

## Data availability statement

The original contributions presented in the study are included in the article/supplementary material, further inquiries can be directed to the corresponding author.

## Author contributions

CZ designed the study. NM collected and analyzed the data. YL performed statistical analysis and interpretation, and made critical revisions to the manuscript. LY, PL, and YX wrote the first draft of the manuscript. All authors contributing to the final version of the manuscript.

## Funding

This work was supported by the National Natural Science Foundation of China with Grant numbers 31671041 and 81971232, Shanghai Municipal Science and Technology Major Project (No. 2018SHZDZX01) and ZJLab.

## Conflict of interest

The authors declare that the research was conducted in the absence of any commercial or financial relationships that could be construed as a potential conflict of interest.

## Publisher’s note

All claims expressed in this article are solely those of the authors and do not necessarily represent those of their affiliated organizations, or those of the publisher, the editors and the reviewers. Any product that may be evaluated in this article, or claim that may be made by its manufacturer, is not guaranteed or endorsed by the publisher.

## Abbreviations

AD, Alzheimer’s disease
Aβ, β amyloid peptide
Akt, protein kinase B
Baf-A1, bafilomycin A1
ISP, insulin signaling pathway
CETSA, cellular thermal shift assay
DMEM, Dulbecco’s modified eagle’s medium
DTT, dithiothreitol; DARTs, drug affinity responsive target stability
GSK3β, glycogen synthase kinase 3
Lona, lonafarnib
LC-3, microtubule-associated protein light chain 3
IR, insulin receptor
IRS-1, insulin recptor substrate 1
mTOR, mammalian target of rapamycin
NFT, neurofibrillary tangles; PBS, phosphate buffered saline
PTEN, phosphatase and tensin homology deleted on chromosome 10
PTau, phosphorylated Tau
PI3K, phosphoinositide 3-kinase
Rhes, ras homologen riched in striatum
SP, senile plague
Tau, Tau protein
WTau, wild-Tau.
